# Presidential Symposium: Transforming Aging Through Technology: A Practical Guide for Early Scholars

**DOI:** 10.1093/geroni/igaf122.1377

**Published:** 2025-12-31

**Authors:** Sohyun Kim, Katherine Britt

## Abstract

The intersection of technology and aging provides opportunities to enhance research, practice, education, and policy. As the global aging population grows, leveraging technology to improve health outcomes, quality of life, and system efficiencies has become increasingly vital. This symposium will explore cutting-edge, technology-driven approaches in aging fields, offering early scholars and professionals practical insights into integrating these innovations into their careers. Speakers will highlight emerging technologies such as artificial intelligence, sensing technology, AI-powered robots, intergenerational technology interventions, and person-centered interventions transforming aging research and practice. Additionally, the session will provide practical guidance on effectively implementing and evaluating technology-based solutions with careful consideration of the needs of older adults across diverse professional settings, including academia, clinical practice, policy development, and community engagement. This symposium aims to empower attendees with actionable strategies to incorporate technology into their work by bridging the gap between technological innovation and its real-world application. Participants will leave with a deeper understanding of how to harness technological advancements to drive progress in aging-related disciplines.

